# Checklist of American sand flies (Diptera, Psychodidae, Phlebotominae): genera, species, and their distribution

**DOI:** 10.3897/zookeys.660.10508

**Published:** 2017-03-08

**Authors:** Paloma Helena Fernandes Shimabukuro, Andrey José de Andrade, Eunice Aparecida Bianchi Galati

**Affiliations:** 1 Centro de Referência Nacional e Internacional para Flebotomíneos, Centro de Pesquisas René Rachou, Fundação Oswaldo Cruz, Av. Augusto de Lima, 1715, Barro Preto, Belo Horizonte, MG, 30190-002, Brazil; 2 Departamento de Patologia Básica, Setor de Ciências Biológicas, Universidade Federal do Paraná, Av. Cel. Francisco H. dos Santos, 210. Jardim das Américas, Curitiba, PR, 81.531-980, Brazil; 3 Departamento de Epidemiologia, Faculdade de Saúde Pública, Universidade de São Paulo, Av. Dr. Arnaldo, 515, Pinheiros, São Paulo, SP, 01246-904, Brazil

**Keywords:** Distribution, Nearctic, Neotropical, Psychodomorpha, sand fly, taxonomy

## Abstract

Phlebotomine sand flies are dipteran insects of medical importance because many species are involved in the transmission of pathogens between human and non-human animals. A total of 530 American species of sand flies is presented in an updated checklist, along with their author(s) and year of publication using the classification by Galati (1995, 2003). Distribution by country is also provided.

## Introduction

Phlebotomine sand flies (Diptera, Psychodidae, Phlebotominae) are medically important insects involved in the transmission of arboviruses, bacteria and protozoan parasites among human and non-human animals (Rangel and Lainson 2009).

There are approximately 1,000 valid described species of sand flies in the world of which 530 are known to occur in the Americas. Historically, the systematics of sand flies has been based on the division of species into few genera (Fairchild 1955, Theodor 1965, Lewis et al. 1977, Young and Duncan 1994). Based on an extensive comparative analysis of characters, Theodor (1965) made an attempt to define groups of American sand flies, but preferred not to give nomenclatorial rank to them, suggesting these groupings might change taxonomic status with future studies. Lewis et al. (1977) considered the Atlantic Ocean as the main cause of differentiation between sand flies from the “Old World” (Paleartic, Afrotropical, Oriental and Australasian regions) and the “New World” (Nearctic and Neotropical regions), and maintained five genera of which two, *Phlebotomus* Rondani & Berté, 1840 and *Sergentomyia* França & Parrot, 1920, occur in the “Old World” and three, *Brumptomyia* França & Parrot, 1921, *Warileya* Hertig, 1948 and *Lutzomyia* França, 1924, are present in the “New World”. Later, Young and Duncan (1994) amended the classification of Lewis et al. (1977), becoming the most widely adopted by those working with sand flies.

Artemiev (1991) attempted to establish a phylogenetic classification for sand flies. He divided sand fly species into two tribes (Idiophlebotomini Artemiev, 1991 and Phlebotomini Rondani, 1840), seven sub-tribes (Idiophlebotomina Artemiev, 1991; Hertigiina Abonnenc & Léger, 1976; Phlebotominae Rondani, 1840; Spelaeomyiina Artemiev, 1991; Sergentomyiina Artemiev, 1991; Australophlebotomina Artemiev, 1991; Brumptomyiina Artemiev, 1991), and 24 genera. However, no explicit method was used to group the sand flies, and his classification was not accepted among researchers of this group of insects.

A proposal for the classification of Phlebotominae was presented by Galati (1995, 2003) who used the cladistic method in her study of American sand flies. In her classification, the tribe Idiophlebotomini Artemiev, 1991 was synonymized with Hertigiini Abonnenc & Léger, 1976, while the tribe Phlebotomini Rondani, 1840 was maintained. Six subtribes from Artemiev (1991) were kept (Idiophlebotomina, Hertigiina, , Sergentomyiina, Australophlebotomina, and Brumptomyiina) and an additional subtribe was created (Psychodopygina Galati, 1995). In addition, one subtribe previously considered synonymous was reinstated (Lutzomyiina Abonnenc & Léger, 1976). Galati reclassified "New World" sand flies into 22 genera. Later, *Edentomyia* Galati, Andrade-Filho, Silva & Falcão, 2003 was proposed as another Neotropical genus in the tribe Phlebotomini; however, this genus was not included in any subtribe (Galati et al. 2003).

The aim of this work is to provide a checklist of valid Phlebotominae species of the Neotropical and Nearctic regions, together with their distribution by country, highlighting their type-locality. This work updates the list of American sand flies and provides currently accepted names for use by taxonomists, students, researchers and health workers.

## Material and methods

The list contains information updated until December, 2016, and data was collated from our own work with sand fly taxonomy, literature surveys, and studies of sand flies deposited in different entomological collections: (i) Coleção de Flebotomíneos (FIOCRUZ/COLFLEB); (ii) Museu de Zoologia da Universidade de São Paulo (MZUSP); (iii) Coleção de Referência da Faculdade de Saúde Pública (FSP–USP); (iv) Coleção Entomológica do Laboratório de Entomologia em Saúde Pública (FSP–USP–LESP–Phlebotominae); (v) Coleção da Seção de Parasitologia do Instituto Butantan (IBut); (vi) Coleção de Flebotomíneos do Instituto Evandro Chagas (COLFlebIEC); (vii) Natural History Museum, London (NHM).

To be considered valid, and therefore included in this checklist, a species name has to meet the criteria of publication in Articles 8 and 9 of International Code of Zoological Nomenclature (ICZN) (1999, 2011).

The checklist presents genera arranged according to the classification of Galati (2003), and subgenera, species groups/series and species are listed alphabetically within each genus. Countries are listed alphabetically and the country of the type locality is mark with asterisk (*). Fossil species are indicated by the symbol †. We intend to add information about synonymies and full references for distributional records in a later, more comprehensive, catalogue.

## Results

This checklist includes 530 species of the Phlebotominae, distributed among 23 genera, occurring in 28 countries.

There are seven “informal” taxa that comprise unavailable names since they do not meet the requirements of the ICZN, and two other names are available but are found to denote more than one taxon (availability of name is not affected according to provisions of the ICZN, Articles 17.2 and 23.8). Here, we report one *nomem dubium* for *Nyssomyia singularis* and one *species inquierenda* for *Bichromomyia inornata*.

Despite the great amount of data on American sand flies and the increased interest in the study of these insects, there has been slow progress in the understanding of taxonomy and systematics of this subfamily. The checklist presented herein aims to give an updated account of which valid species have been recorded in the Neotropical and Neartic regions, as well as provide information on the geographical range of these species by country.

## Systematics

**Phylum Arthropoda von Siebold, 1848**

**Subphylum Hexapoda Latreille, 1825**

**Class Insecta Linnaeus, 1758**

**Order Diptera Linnaeus, 1758**

**Sub-order Psychodomorpha Hennig, 1968**

**Family Psychodidae Newman, 1834**

**Subfamily Phlebotominae Rondani, 1840**

**Tribe Hertigiini Abonnenc & Léger, 1976**

**Subtribe HERTIGIINA Abonnenc & Léger, 1976**

**Genus *Hertigia* Fairchild, 1949**

***Hertigia hertigi* Fairchild, 1949**

**Distribution.** Costa Rica, Panama*.

**Genus *Warileya* Hertig, 1948**

***Warileya euniceae* Fernández, Carbajal, Astete & Wooster, 1998**

**Distribution.** Peru*.

***Warileya fourgassiensis* Le Pont & Desjeux, 1984**

**Distribution.** French Guiana*.

***Warileya leponti* Galati & Cáceres, 1999**

**Distribution.** Peru*.

***Warileya lumbrerasi* Ogosuku, Perez, Davies & Villaseca, 1996**

**Distribution.** Peru*.

***Warileya nigrosaccula* Fairchild & Hertig, 1951**

**Distribution.** Colombia, Panama*.

***Warileya phlebotomanica* Hertig, 1948**

**Distribution.** Ecuador, Peru*.

***Warileya rotundipennis* Fairchild & Hertig, 1951**

**Distribution.** Bolivia, Colombia, Costa Rica, Panama*, Peru.

***Warileya yungasi* Velasco & Trapido, 1974**

**Distribution.** Bolivia*.

**Tribe Phlebotomini Rondani, 1840**

**Subtribe Brumptomyiina Artemiev, 1991**

**Genus *Brumptomyia* França and Parrot, 1921**

***Brumptomyia angelae* Galati, Santos & Silva, 2007**

**Distribution.** Brazil*.

***Brumptomyia avellari* (Costa Lima, 1932)**

**Distribution.** Argentina, Bolivia, Brazil*, Colombia, Panama, Paraguay, Peru, Venezuela.

***Brumptomyia beaupertuyi* (Ortiz, 1954)**

**Distribution.** Colombia, Venezuela*.

***Brumptomyia bragai* Mangabeira & Sherlock, 1961**

**Distribution.** Brazil*.

***Brumptomyia brumpti* (Larrousse, 1920)**

**Distribution.** Argentina, Bolivia, Brazil*.

***Brumptomyia cardosoi* (Barretto & Coutinho, 1941)**

**Distribution.** Brazil*.

***Brumptomyia carvalheiroi* Shimabukuro, Marassá & Galati, 2007**

**Distribution.** Brazil*.

***Brumptomyia cunhai* (Mangabeira, 1942)**

**Distribution.** Brazil*, Honduras.

***Brumptomyia devenanzii* (Ortiz & Scorza, 1963)**

**Distribution.** Venezuela*.

***Brumptomyia figueireidoi* Mangabeira & Sherlock, 1961**

**Distribution.** Brazil*.

***Brumptomyia galindoi* (Fairchild & Hertig, 1947)**

**Distribution.** Brazil, Panama*.

***Brumptomyia guimaraesi* (Coutinho & Barretto, 1941)**

**Distribution.** Argentina, Brazil*, Paraguay.

***Brumptomyia hamata* (Fairchild & Hertig, 1947)**

**Distribution.** Belize, Colombia, Ecuador, Mexico, Panama*, Peru.

***Brumptomyia leopoldoi* (Rodriguez, 1953)**

**Distribution.** Belize, Colombia, Ecuador*, Panama.

***Brumptomyia mangabeirai* (Barretto & Coutinho, 1941)**

**Distribution.** Brazil*.

***Brumptomyia mesai* Sherlock, 1962**

**Distribution.** Belize, Colombia*, Honduras, Mexico.

***Brumptomyia nitzulescui* (Costa Lima, 1932)**

**Distribution.** Brazil*.

***Brumptomyia orlandoi* Fraiha, Shaw & Lainson, 1970**

**Distribution.** Brazil*.

***Brumptomyia ortizi* Martins, Silva & Falcão, 1971**

**Distribution.** Brazil*.

***Brumptomyia pentacantha* (Barretto, 1947)**

**Distribution.** Bolivia, Brazil*, Colombia, Ecuador, Peru.

***Brumptomyia pintoi* (Costa Lima, 1932**)

**Distribution.** Argentina, Bolivia, Brazil*, French Guiana, Surinam, Venezuela.

***Brumptomyia quimperi* Galati & Cáceres, 1999**

**Distribution.** Peru*.

***Brumptomyia spinosipes* (Floch & Abonnenc, 1943)**

**Distribution.** Brazil, French Guiana*, Panama.

***Brumptomyia travassosi* (Mangabeira, 1942)**

**Distribution.** Brazil*, French Guiana, Panama, Surinam.

***Brumptomyia troglodytes* (Lutz, 1922)**

**Distribution.** Brazil*, Peru.

***Brumptomyia virgensi* Mangabeira & Sherlock, 1961**

**Distribution.** Brazil*.

**Genus *Oligodontomyia* Galati, 1995**

***Oligodontomyia isopsi* (Léger & Ferte, 1996)**

**Distribution.** Chile*.

***Oligodontomyia oligodonta* (Young, Pérez & Romero, 1985)**

**Distribution.** Peru*.

***Oligodontomyia toroensis* (Le Pont, Torrez-Espejo & Dujardin, 1997)**

**Distribution.** Bolivia*.

**Subtribe Sergentomyiina Artemiev, 1991**

**Genus *Deanemyia* Galati, 1995**

***Deanemyia appendiculata* (Martins, Falcão & Silva, 1961)**

**Distribution.** Brazil*.

***Deanemyia derelicta* (Freitas & Barrett, 1999)**

**Distribution.** Brazil*.

***Deanemyia maruaga* (Alves, Freitas & Barrett, 2008)**

**Distribution.** Brazil*.

***Deanemyia ramirezi* (Martins, Falcão, Silva & Miranda-Filho, 1982)**

**Distribution.** Bolivia, Brazil*.

**Note.** The record for Bolivia is based on specimens collected in Aguas Calientes Department of Santa Cruz and donated by François Le Pont to one of us (EABG).

***Deanemyia samueli*** (Deane, 1955)

**Distribution.** Brazil*.

**Genus *Micropygomyia* Barretto, 1962**

**Subgenus (*Coquillettimyia*) Galati, 1995**

**Series chiapanensis Theodor, 1965**

**Micropygomyia (Coquillettimyia) californica (Fairchild & Hertig, 1957)**

**Distribution.** United States of America*.

**Micropygomyia (Coquillettimyia) chiapanensis (Dampf, 1947)**

**Distribution.** Costa Rica, El Salvador, Honduras, Mexico*, Nicaragua, Panama.

**Micropygomyia (Coquillettimyia) stewarti (Mangabeira & Galindo, 1944)**

**Distribution.** Mexico, United States of America*.

**Series vexator Fairchild, 1955**

**Micropygomyia (Coquillettimyia) apache (Young & Perkins, 1984)**

**Distribution.** United States of America*.

**Micropygomyia (Coquillettimyia) oppidana (Dampf, 1944)**

**Distribution.** Canada, Mexico*, United States of America.

**Micropygomyia (Coquillettimyia) vexator (Coquillett, 1907)**

**Distribution.** Canada, Mexico, United States of America*.

**Micropygomyia (Coquillettimyia) vindicator (Dampf, 1944)**

**Distribution.** Mexico*.

**Subgenus (*Micropygomyia*) Barretto, 1962**

**Series cayennensis Fairchild, 1955**

**Micropygomyia (Micropygomyia) absonodonta (Feliciangeli, 1995)**

**Distribution.** Peru, Venezuela*.

**Micropygomyia (Micropygomyia) ancashensis Galati & Cáceres, 2007**

**Distribution.** Peru*.

**Micropygomyia (Micropygomyia) cayennensis cayennensis (Floch & Abonnenc, 1941)**

**Distribution.** Belize, Brazil, Colombia, Costa Rica, Ecuador, El Salvador, French Guiana*, Honduras, Mexico, Nicaragua, Panama, Peru, Trinidad and Tobago, Venezuela.

**Micropygomyia (Micropygomyia) cayennensis braci (Lewis, 1967)**

**Distribution.** Cayman Islands*.

**Micropygomyia (Micropygomyia) cayennensis cruzi (Gonzáles & García, 1981)**

**Distribution.** Cuba*.

**Micropygomyia (Micropygomyia) cayennensis hispaniolae (Fairchild & Trapido, 1950)**

**Distribution.** Dominican Republic*, Haiti.

**Micropygomyia (Micropygomyia) cayennensis jamaicensis (Fairchild & Trapido, 1950)**

**Distribution.** Jamaica*.

**Micropygomyia (Micropygomyia) cayennensis maciasi (Fairchild & Hertig, 1948)**

**Distribution.** Belize, Guatemala, Mexico*.

**Micropygomyia (Micropygomyia) cayennensis puertoricensis (Fairchild & Hertig, 1948)**

**Distribution.** Puerto Rico*.

**Micropygomyia (Micropygomyia) cayennensis viequesensis (Fairchild & Hertig, 1948)**

**Distribution.** Puerto Rico*, Virgin Islands.

**Micropygomyia (Micropygomyia) ctenidophora (Fairchild & Hertig, 1948)**

**Distribution.** Mexico*.

**Micropygomyia (Micropygomyia) cubensis (Fairchild & Trapido, 1950)**

**Distribution.** Cuba*, United States of America.

**Micropygomyia (Micropygomyia) duppyorum (Fairchild & Trapido, 1950)**

**Distribution.** Jamaica*.

**Micropygomyia (Micropygomyia) durani (Vargas & Diaz-Nájera, 1952)**

**Distribution.** El Salvador, Honduras, Mexico*.

**Micropygomyia (Micropygomyia) lewisi (Feliciangeli, Ordoñez & Fernández, 1984)**

**Distribution.** Venezuela*.

**Micropygomyia (Micropygomyia) micropyga (Mangabeira, 1942)**

**Distribution.** Bolivia, Brazil*, Colombia, Costa Rica, Ecuador, French Guiana, Panama, Peru, Trinidad and Tobago, Venezuela.

**Micropygomyia (Micropygomyia) schreiberi (Martins, Falcão & Silva, 1975)**

**Distribution.** Brazil*.

**Micropygomyia (Micropygomyia) yencanensis (Ortiz, 1965)**

**Distribution.** Colombia, Venezuela*.

**Series pilosa Theodor, 1965**

**Micropygomyia (Micropygomyia) chassigneti (Floch & Abonnenc, 1944)**

**Distribution.** Brazil, Colombia, French Guiana*, Surinam.

**Micropygomyia (Micropygomyia) mangabeirana (Martins, Falcão & Silva, 1963)**

**Distribution.** Brazil*.

**Micropygomyia (Micropygomyia) pilosa (Damasceno & Causey, 1944)**

**Distribution.** Brazil*, Colombia, Costa Rica, French Guiana, Grenada, Panama, Trinidad and Tobago, Venezuela.

**Subgenus (*Sauromyia*) Artemiev, 1991**

**Series atroclavata Fairchild, 1955**

**Micropygomyia (Sauromyia) atroclavata (Knab, 1913)**

**Distribution.** Colombia, Costa Rica, Guadeloupe, Martinica, Panama, Trinidad and Tobago*, Venezuela, Virgin Islands.

**Micropygomyia (Sauromyia) venezuelensis (Floch & Abonnenc, 1948)**

**Distribution.** Colombia, Venezuela*.

**Series oswaldoi Barretto, 1962**

**Micropygomyia (Sauromyia) capixaba (Dias, Falcão, Silva & Martins, 1987)**

**Distribution.** Brazil*.

**Micropygomyia (Sauromyia) dereuri (Le Pont, Matias, Martinez & Dujardin, 2004)**

**Distribution.** Bolivia*.

†**Micropygomyia (Sauromyia) dorafeliciangeli Andrade-Filho, Galati & Brazil, 2009**

**Distribution.** Dominican amber*.

**Micropygomyia (Sauromyia) ferreirana (Barretto, Martins & Pellegrino, 1956)**

**Distribution.** Brazil*.

**Micropygomyia (Sauromyia) huacalquensis (Le Pont, Matias, Martinez & Dujardin, 2004)**

**Distribution.** Bolivia*.

**Micropygomyia (Sauromyia) longipennis (Barretto, 1946)**

**Distribution.** Brazil*, Peru.

**Micropygomyia (Sauromyia) machupicchu (Martins, Llanos & Silva, 1975)**

**Distribution.** Peru*.

**Micropygomyia (Sauromyia) oswaldoi (Mangabeira, 1942)**

**Distribution.** Argentina, Bolivia, Brazil*.

†**Micropygomyia (Sauromyia) paterna (Quate, 1963)**

**Distribution.** Mexican amber*.

**Micropygomyia (Sauromyia) peresi (Mangabeira, 1942)**

**Distribution.** Argentina, Bolivia, Brazil*, French Guiana.

**Micropygomyia (Sauromyia) petari Galati, Marassá & Gonçalves-Andrade, 2003**

**Distribution.** Brazil*.

**Micropygomyia (Sauromyia) pratti (Vargas & Diaz-Nájera, 1951)**

**Distribution.** Mexico*.

**Micropygomyia (Sauromyia) pusilla (Dias, Martins, Falcão & Silva, 1986)**

**Distribution.** Brazil*, French Guiana.

**Micropygomyia (Sauromyia) quechua (Martins, Llanos & Silva, 1975)**

**Distribution.** Peru*.

**Micropygomyia (Sauromyia) quinquefer (Dyar, 1929)**

**Distribution.** Argentina*, Bolivia, Brazil.

**Micropygomyia (Sauromyia) rorotaensis (Floch & Abonnenc, 1944)**

**Distribution.** Brazil, Colombia, French Guiana*, Peru, Surinam, Panama, Venezuela.

**Micropygomyia (Sauromyia) saccai (Feliciangeli, Ramírez Pérez & Ramírez, 1989)**

**Distribution.** Venezuela*.

**Micropygomyia (Sauromyia) trinidadensis (Newstead, 1922)**

**Distribution.** Belize, Bolivia, Brazil, Colombia, Costa Rica, Ecuador, El Salvador, French Guiana, Guatemala, Honduras, Mexico, Nicaragua, Peru, Panama, Surinam, Trinidad and Tobago*, Venezuela.

**Micropygomyia (Sauromyia) villelai (Mangabeira, 1942)**

**Distribution.** Brazil*.

**Micropygomyia (Sauromyia) vonatzingeni Galati, 2007**

**Distribution.** Brazil*.

**Micropygomyia (Sauromyia) zikani (Barretto, 1950)**

**Distribution.** Brazil*.

**Subgenus (*Silvamyia*) Galati, 1995**

**Micropygomyia (Silvamyia) acanthopharynx (Martins, Falcão & Silva, 1962)**

**Distribution.** Brazil*.

**Micropygomyia (Silvamyia) echinatopharynx Andrade-Filho, Galati, Andrade & Falcão, 2004**

**Distribution.** Brazil*.

### *Micropygomyia incertae sedis*

†***Micropygomyia brandaoi* Andrade-Filho, Galati, Falcão & Brazil, 2008**

**Distribution.** Dominican amber*.

***Micropygomyia xerophila* (Young, Brener & Wargo, 1983)**

**Distribution.** United States of America*.

**Subtribe Lutzomyiina Abonnenc and Léger, 1976**

**Genus *Sciopemyia* Barretto, 1962**

***Sciopemyia fluviatilis* (Floch & Abonnenc, 1944)**

**Distribution.** Brazil, French Guiana*.

***Sciopemyia microps* (Mangabeira, 1942)**

**Distribution.** Brazil*.

***Sciopemyia nematoducta* (Young & Arias, 1984)**

**Distribution.** Brazil*, Colombia.

***Sciopemyia pennyi* (Arias & Freitas, 1981)**

**Distribution.** Brazil*.

***Sciopemyia preclara* (Young & Arias, 1984)**

**Distribution.** Bolivia, Brazil, Colombia*, Peru.

***Sciopemyia servulolimai* (Damasceno & Causey, 1945)**

**Distribution.** Bolivia, Brazil*, Colombia, Peru.

***Sciopemyia sordellii* (Shannon & Del Ponte, 1927)**

**Distribution.** Argentina*, Bolivia, Brazil, Colombia, Costa Rica, Ecuador, French Guiana, Panama, Peru, Trinidad and Tobago, Venezuela.

***Sciopemyia vattierae* (Le Pont & Desjeux, 1992)**

**Distribution.** Bolivia*, Colombia, Peru.

**Genus *Lutzomyia* França, 1924**

**Subgenus (*Castromyia*) Mangabeira, 1942**

**Lutzomyia (Castromyia) amarali (Barretto & Coutinho, 1940)**

**Distribution.** Brazil*.

**Lutzomyia (Castromyia) caligata Martins, Falcão & Silva, 1965**

**Distribution.** Brazil*.

**Lutzomyia (Castromyia) castroi (Barretto & Coutinho, 1941)**

**Distribution.** Brazil*.

**Subgenus (*Helcocyrtomyia*) Barretto, 1962**

**Series osornoi Galati & Cáceres, 1994**

**Lutzomyia (Helcocyrtomyia) caballeroi Blancas, Cáceres & Galati, 1989**

**Distribution.** Peru*.

**Lutzomyia (Helcocyrtomyia) castanea Galati & Cáceres, 1994**

**Distribution.** Ecuador, Peru*.

**Lutzomyia (Helcocyrtomyia) ceferinoi (Ortiz & Alvarez, 1963)**

**Distribution.** Colombia, Venezuela*.

**Lutzomyia (Helcocyrtomyia) erwindonaldoi (Ortiz, 1978)**

**Distribution.** Colombia, Venezuela*.

**Lutzomyia (Helcocyrtomyia) herreri Galati & Cáceres, 2003**

**Distribution.** Peru*.

**Lutzomyia (Helcocyrtomyia) imperatrix (Alexander, 1944)**

**Distribution.** Peru*.

**Lutzomyia (Helcocyrtomyia) larensis Arredondo, 1987**

**Distribution.** Venezuela*.

**Lutzomyia (Helcocyrtomyia) munaypata Ogusuku, Chevarria, Porras & Pérez, 1999**

**Distribution.** Peru*.

**Lutzomyia (Helcocyrtomyia) osornoi (Ristorcelli & Van Ty, 1941)**

**Distribution.** Bolivia, Colombia*, Ecuador, Peru.

**Lutzomyia (Helcocyrtomyia) quillabamba Ogusuku, Chevarria, Porras & Pérez, 1999**

**Distribution.** Peru*.

**Lutzomyia (Helcocyrtomyia) rispaili Torrez-Espejo, Cáceres & Le Pont, 1995**

**Distribution.** Bolivia*, Peru.

**Lutzomyia (Helcocyrtomyia) strictivilla Young, 1979**

**Distribution.** Colombia*, Ecuador, Venezuela.

**Lutzomyia (Helcocyrtomyia) wattsi Fernández, Carbajal, Astete & Wooster, 1998**

**Distribution.** Peru*.

**Series peruensis Barretto, 1962**

**Lutzomyia (Helcocyrtomyia) ayacuchensis Cáceres & Galati, 1988**

**Distribution.** Ecuador, Peru*.

**Lutzomyia (Helcocyrtomyia) blancasi Galati & Cáceres, 1990**

**Distribution.** Peru*.

**Lutzomyia (Helcocyrtomyia) chavinensis Pérez & Ogusuku, 1999**

**Distribution.** Peru*.

**Lutzomyia (Helcocyrtomyia) galatiae Le Pont, Martínez, Torrez-Espejo & Dujardin, 1998**

**Distribution.** Bolivia*.

**Lutzomyia (Helcocyrtomyia) noguchii (Shannon, 1929)**

**Distribution.** Peru*.

**Lutzomyia (Helcocyrtomyia) pallidithorax Galati & Cáceres, 1994**

**Distribution.** Peru*.

**Lutzomyia (Helcocyrtomyia) peruensis (Shannon, 1929)**

**Distribution.** Bolivia, Peru*.

**Lutzomyia (Helcocyrtomyia) pescei (Hertig, 1943)**

**Distribution.** Peru*.

**Lutzomyia (Helcocyrtomyia) tejadai Galati & Cáceres, 1990**

**Distribution.** Peru*.

**Series sanguinaria Barretto, 1962**

**Lutzomyia (Helcocyrtomyia) adamsi Fernández, Galati, Carbajal, Wooster & Watts, 1998**

**Distribution.** Peru*.

**Lutzomyia (Helcocyrtomyia) botella (Fairchild & Hertig, 1961)**

**Distribution.** Panama*.

**Lutzomyia (Helcocyrtomyia) caceresi Le Pont, Matías, Martínez & Dujardin, 2004**

**Distribution.** Bolivia*.

**Lutzomyia (Helcocyrtomyia) cirrita Young & Porter, 1974**

**Distribution.** Colombia*.

**Lutzomyia (Helcocyrtomyia) gonzaloi Ogusuku, Canales & Pérez, 1997**

**Distribution.** Peru*.

**Lutzomyia (Helcocyrtomyia) guderiani Torrez-Espejo, Cáceres & Le Pont, 1995**

**Distribution.** Bolivia*, Peru.

**Lutzomyia (Helcocyrtomyia) hartmanni (Fairchild & Hertig, 1957)**

**Distribution.** Colombia, Costa Rica, Ecuador, Mexico, Panama*, Peru.

**Lutzomyia (Helcocyrtomyia) kirigetiensis Galati & Cáceres, 1992**

**Distribution.** Peru*.

**Lutzomyia (Helcocyrtomyia) monzonensis Ogusuku, Canales & Pérez, 1997**

**Distribution.** Peru*.

**Lutzomyia (Helcocyrtomyia) sanguinaria (Fairchild & Hertig, 1957)**

**Distribution.** Colombia, Costa Rica, Ecuador, Honduras, Nicaragua, Panama*, Peru.

**Lutzomyia (Helcocyrtomyia) scorzai (Ortiz, 1965)**

**Distribution.** Colombia*, Peru, Venezuela.

**Lutzomyia (Helcocyrtomyia) tolimensis Carrasquilla, Munstermann, Marín, Ocampo & Ferro, 2012**

**Distribution.** Colombia*.

**Lutzomyia (Helcocyrtomyia) tortura Young & Rogers, 1984**

**Distribution.** Bolivia, Colombia, Ecuador*.

**Lutzomyia (Helcocyrtomyia) velezi Bejarano, Vivero & Uribe, 2010**

**Distribution.** Colombia*.

**Subgenus (*Lutzomyia*) França, 1924**

**Lutzomyia (Lutzomyia) alencari Martins, Souza & Falcão, 1962**

**Distribution.** Brazil*.

**Lutzomyia (Lutzomyia) almerioi Galati & Nunes, 1999**

**Distribution.** Brazil*.

**Lutzomyia (Lutzomyia) battistinii (Hertig, 1943)**

**Distribution.** Brazil, Peru*.

**Lutzomyia (Lutzomyia) bicornuta (Blancas & Herrer, 1960)**

**Distribution.** Peru*.

**Lutzomyia (Lutzomyia) bifoliata Osorno-Mesa, Morales, Osorno & Hoyos, 1970**

**Distribution.** Colombia*.

**Lutzomyia (Lutzomyia) cavernicola (Costa Lima, 1932)**

**Distribution.** Brazil*.

**Lutzomyia (Lutzomyia) cruzi (Mangabeira, 1938)**

**Distribution.** Bolivia, Brazil*.

**Lutzomyia (Lutzomyia) dispar Martins & Silva, 1963**

**Distribution.** Brazil*.

**Lutzomyia (Lutzomyia) elizabethrangelae Vilela, Azevedo & Godoy, 2015**

**Distribution.** Brazil*.

**Lutzomyia (Lutzomyia) falquetoi Pinto & Santos, 2007**

**Distribution.** Brazil*.

**Lutzomyia (Lutzomyia) fonsecai (Costa Lima, 1932)**

**Distribution.** Bolivia*.

**Note.** Placement in *Lutzomyia* based on the study by one of us (EABG) of specimens collected in several caves close to the type-locality of *Lutzomyia fonsecai* in the Chiquitano seasonally dry forest of Serrania Santiago (Calvario of the Robore municipality and Aguas Calientes near Robore, Santa Cruz Department).

**Lutzomyia (Lutzomyia) forattinii Galati, Rego, Nunes & Teruya, 1985**

**Distribution.** Bolivia, Brazil*.

**Lutzomyia (Lutzomyia) gaminarai (Cordero, Vogelsang & Cossio, 1928)**

**Distribution.** Brazil, Uruguay*.

**Lutzomyia (Lutzomyia) ischnacantha Martins, Souza & Falcão, 1962**

**Distribution.** Brazil*.

**Lutzomyia (Lutzomyia) ischyracantha Martins, Falcão & Silva, 1962**

**Distribution.** Brazil*.

**Lutzomyia (Lutzomyia) lichyi (Floch & Abonnenc, 1950)**

**Distribution.** Brazil, Colombia, Costa Rica, Ecuador, French Guiana, Panama, Peru, Trinidad and Tobago, Venezuela*.

**Lutzomyia (Lutzomyia) longipalpis (Lutz & Neiva, 1912)**

**Distribution.** Argentina, Bolivia, Brazil*, Colombia, Costa Rica, El Salvador, Guatemala, Honduras, Mexico, Nicaragua, Panama, Paraguay, Uruguay, Venezuela.

**Lutzomyia (Lutzomyia) matiasi Le Pont & Mollinedo, 2009**

**Distribution.** Bolivia*.

**Lutzomyia (Lutzomyia) pseudolongipalpis Arrivillaga & Feliciangeli, 2001**

**Distribution.** Venezuela*.

**Lutzomyia (Lutzomyia) renei (Martins, Falcão & Silva, 1957)**

**Distribution.** Brazil*.

**Lutzomyia (Lutzomyia) souzalopesi Martins, Silva & Falcão, 1970**

**Distribution.** Brazil*.

**Subgenus (*Tricholateralis*) Galati, 1995**

**Lutzomyia (Tricholateralis) araracuarensis Morales & Minter, 1981**

**Distribution.** Brazil, Colombia*.

**Lutzomyia (Tricholateralis) carvalhoi (Damasceno, Causey & Arouck, 1945)**

**Distribution.** Brazil*, French Guiana.

**Lutzomyia (Tricholateralis) cruciata (Coquillett, 1907)**

**Distribution.** Belize, Brazil, Costa Rica, El Salvador, Guatemala*, Honduras, Mexico, Nicaragua, Panama, United States of America.

**Lutzomyia (Tricholateralis) cultellata Freitas & Albuquerque, 1996**

**Distribution.** Brazil*, Peru.

**Note.** Placement is his subgenus was possible due to the study of specimens provided to us (EABG and PHFS) by Mr. Rui Freitas (Instituto Nacional de Pesquisas da Amazônia). We concluded it belongs to the subgenus Tricholateralis because among other characters this species presents the ventro-cervical sensillae, setae in the abdominal pleura and lacks the ascoids with posterior spurs.

**Lutzomyia (Tricholateralis) diabolica (Hall, 1936)**

**Distribution.** Mexico, United States of America*.

**Lutzomyia (Tricholateralis) evangelistai Martins & Fraiha, 1971**

**Distribution.** Bolivia, Brazil*, Colombia, Peru.

**Lutzomyia (Tricholateralis) falcata Young, Morales & Ferro, 1994**

**Distribution.** Brazil, Colombia*, Ecuador.

**Lutzomyia (Tricholateralis) flabellata Martins & Silva, 1964**

**Distribution.** Bolivia, Brazil*.

**Lutzomyia (Tricholateralis) gomezi (Nitzulescu, 1931)**

**Distribution.** Bolivia, Brazil, Colombia, Costa Rica, Ecuador, El Salvador, French Guiana, Honduras, Mexico, Nicaragua, Panama, Peru, Trinidad and Tobago, Venezuela*.

**Lutzomyia (Tricholateralis) legerae Le Pont, Gantier, Hue & Valle, 1995**

**Distribution.** Nicaragua*.

**Lutzomyia (Tricholateralis) maesi Le Pont, Ibáñez–Bernal & Fuentes, 2011**

**Distribution.** Nicaragua*.

**Lutzomyia (Tricholateralis) marinkellei Young, 1979**

**Distribution.** Brazil*, Colombia.

**Lutzomyia (Tricholateralis) sherlocki Martins, Silva & Falcão, 1971**

**Distribution.** Bolivia, Brazil*, Colombia, Ecuador, Peru.

**Lutzomyia (Tricholateralis) spathotrichia Martins, Falcão & Silva, 1963**

**Distribution.** Bolivia, Brazil*, Ecuador, French Guiana.

***Lutzomyia incertae sedis***

***Lutzomyia chotensis* Galati, Cáceres & Zorilla, 2003**

**Distribution.** Peru*.

***Lutzomyia ignacioi* (Young, 1972)**

**Distribution.** Colombia, Venezuela*.

**Note.** Galati (2003) placed this species in *Psathryromyia* as *incertae sedis*. However, Sábio, PB (pers. comm.) examined the type material deposited in the Entomological Collection - Smithsonian Institution / Walter Reeed Biosystematic Unit, Suitland, MD - USA) and observed the presence of the ventro-cervical sensillae and the papilla in F3, the setae in the anterior region of the katepisternum is absent, ascoids present reduced posterior spurs and spermathecae are ringed. These characters are synapomorphies shared by some species of *Lutzomyia* (*Castromyia*, *Tricholateralis* and *Lutzomyia*), but this species also lacks characters to placed it with confidence in any of these three subgenera.

***Lutzomyia infusca* Porter & Young, 1999**

**Distribution.** Guatemala*.

***Lutzomyia manciola* Ibáñez-Bernal, 2001**

**Distribution.** Belize*.

**Note.** The insertion of this species in *Lutzomyia* is provisional. There were no female characters to be observed that could lead to more accurate placement in any genus, nor was the male known. The inclusion of *Lutzomyia manciola* in *Sciopemyia* was suggested by Ibáñez-Bernal (2001). However, *Lutzomyia manciola* do not present the head and labrum-epipharynx shorter than the sum of flagellomeres FI + FII, which are diagnostic characters for *Sciopemyia*.

***Lutzomyia ponsi* (Perruollo, 1984)**

**Distribution.** Venezuela*.

**Note.** The description of this species does not provide sufficient information to place it in any genus; and the similarity of their spermathecae with those of *Lutzomyia ignacioi* led us to include it together with this species in the genus *Lutzomyia*.

***Lutzomyia tanyopsis* Young & Perkins, 1984**

**Distribution.** United States of America*.

***Lutzomyia vargasi* (Fairchild & Hertig, 1961)**

**Distribution.** Mexico*.

**Genus *Migonemyia* Galati, 1995**

**Subgenus (*Blancasmyia*) Galati, 1995**

**Migonemyia (Blancasmyia) bursiformis (Floch & Abonnenc, 1944)**

**Distribution.** Brazil, Colombia, Ecuador, French Guiana*, Venezuela.

**Migonemyia (Blancasmyia) cerqueirai (Causey & Damasceno, 1945)**

**Distribution.** Brazil*, Colombia, Peru.

**Migonemyia (Blancasmyia) gorbitzi (Blancas, 1960)**

**Distribution.** Colombia, Costa Rica, Ecuador, Panama, Peru*.

**Migonemyia (Blancasmyia) moucheti (Pajot & Le Pont, 1978)**

**Distribution.** Brazil, French Guiana*, Peru.

**Subgenus (*Migonemyia*) Galati, 1995**

**Migonemyia (Migonemyia) migonei (França, 1920)**

**Distribution.** Argentina, Bolivia, Brazil, Colombia, Paraguay*, Peru, Trinidad and Tobago, Venezuela.

**Migonemyia (Migonemyia) rabelloi (Galati & Gomes, 1992)**

**Distribution.** Brazil*.

**Migonemyia (Migonemyia) vaniae Galati, Fonseca & Marassá, 2007**

**Distribution.** Brazil*.

**Genus *Pintomyia* Costa Lima, 1932**

**Subgenus** (***Pifanomyia*) Ortiz and Scorza, 1963**

**Series evansi Galati, 1995**

**Pintomyia (Pifanomyia) evansi (Nuñez-Tovar, 1924)**

**Distribution.** Colombia, Costa Rica, El Salvador, Guatemala, Honduras, Mexico, Nicaragua, Peru, Venezuela*.

**Pintomyia (Pifanomyia) maranonensis (Galati, Cáceres & Le Pont, 1995)**

**Distribution.** Ecuador, Peru*.

**Pintomyia (Pifanomyia) nevesi (Damasceno & Arouck, 1956)**

**Distribution.** Bolivia, Brazil*, Colombia, Ecuador, Peru.

**Pintomyia (Pifanomyia) ovallesi (Ortiz, 1952)**

**Distribution.** Belize, Colombia, Costa Rica, Guatemala, Honduras, Mexico, Nicaragua, Panama, Trinidad and Tobago, Venezuela*.

**Series monticola Artemiev, 1991**

**Pintomyia (Pifanomyia) misionensis (Castro, 1959**)

**Distribution.** Argentina*, Brazil, Paraguay.

**Pintomyia (Pifanomyia) monticola (Costa Lima, 1932)**

**Distribution.** Argentina, Brazil*, Paraguay, Peru.

**Series pacae Galati, 1995**

**Pintomyia (Pifanomyia) gruta (Ryan, 1986)**

**Distribution.** Brazil*.

**Pintomyia (Pifanomyia) pacae (Floch & Abonnenc, 1943)**

**Distribution.** Brazil, French Guiana*, Surinam.

**Series pia Galati, 1995**

**Pintomyia (Pifanomyia) emberai (Bejarano, Duque & Vélez, 2004)**

**Distribution.** Colombia*.

**Pintomyia (Pifanomyia) limafalcaoae Wolff & Galati, 2002**

**Distribution.** Colombia*.

**Pintomyia (Pifanomyia) pia (Fairchild & Hertig, 1961)**

**Distribution.** Bolivia, Colombia, Costa Rica, Panama*, Peru, Venezuela.

**Pintomyia (Pifanomyia) reclusa (Fernández & Rogers, 1991)**

**Distribution.** Peru*.

**Pintomyia (Pifanomyia) suapiensis (Le Pont, Torrez-Espejo & Dujardin, 1997)**

**Distribution.** Bolivia*, Peru.

**Pintomyia (Pifanomyia) tihuiliensis (Le Pont, Torrez-Espejo & Dujardin, 1997)**

**Distribution.** Bolivia*, Colombia, Peru.

**Pintomyia (Pifanomyia) tocaniensis (Le Pont, Torrez-Espejo & Dujardin, 1997)**

**Distribution.** Bolivia*, Peru.

**Pintomyia (Pifanomyia) torrealbai (Martins, Fernandez & Falcão, 1979)**

**Distribution.** Venezuela*.

**Pintomyia (Pifanomyia) valderramai (Cazorla, 1988)**

**Distribution.** Venezuela*.

**Series serrana Barretto, 1962**

**Pintomyia (Pifanomyia) boliviana (Velasco & Trapido, 1974)**

**Distribution.** Bolivia*.

**Pintomyia (Pifanomyia) christophei (Fairchild & Trapido, 1950)**

**Distribution.** Dominican Republic*, Haiti.

**Pintomyia (Pifanomyia) diazi (Gonzales & Garcia, 1981)**

**Distribution.** Cuba*.

**Pintomyia (Pifanomyia) guilvardae (Le Pont, Martinez, Torrez-Espejo & Dujardin, 1998)**

**Distribution.** Bolivia*.

**Pintomyia (Pifanomyia) novoae (Gonzales & Garcia, 1981)**

**Distribution.** Cuba*.

**Pintomyia (Pifanomyia) odax (Fairchild & Hertig, 1961)**

**Distribution.** Brazil, Costa Rica, French Guiana, Guatemala, Honduras, Nicaragua, Panama*, Venezuela.

**Pintomyia (Pifanomyia) oresbia (Fairchild & Hertig, 1961)**

**Distribution.** Costa Rica, Panama*.

**Pintomyia (Pifanomyia) orestes (Fairchild & Trapido, 1950)**

**Distribution.** Brazil, Cayman Islands, Cuba*.

**Pintomyia (Pifanomyia) ottolinai (Ortiz & Scorza, 1963)**

**Distribution.** Venezuela*.

**Pintomyia (Pifanomyia) piedraferroi (León, 1971)**

**Distribution.** Guatemala*.

**Pintomyia (Pifanomyia) robusta (Galati, Cáceres & Le Pont, 1995)**

**Distribution.** Ecuador, Peru*.

**Pintomyia (Pifanomyia) serrana (Damasceno & Arouck, 1949)**

**Distribution.** Belize, Bolivia, Brazil*, Colombia, Costa Rica, French Guiana, Guatemala, Honduras, Mexico, Nicaragua, Panama, Peru, Venezuela.

**Pintomyia (Pifanomyia) torresi (Le Pont & Desjeux, 1991)**

**Distribution.** Argentina, Bolivia*.

**Series townsendi Galati, 1995**

**Pintomyia (Pifanomyia) amilcari (Arredondo, 1984)**

**Distribution.** Venezuela*.

**Pintomyia (Pifanomyia) longiflocosa (Osorno-Mesa, Morales, Osorno & Hoyos, 1970)**

**Distribution.** Colombia*.

**Pintomyia (Pifanomyia) nadiae (Feliciangeli, Arredondo & Ward, 1992)**

**Distribution.** Venezuela*.

† **Pintomyia (Pifanomyia) paleotownsendi Andrade-Filho, Falcão, Galati & Brazil, 2006**

**Distribution.** Dominican amber*.

† **Pintomyia (Pifanomyia) paloetrichia Andrade-Filho, Brazil, Falcão & Galati, 2007**

**Distribution.** Dominican amber*.

**Pintomyia (Pifanomyia) quasitownsendi (Osorno, Osorno-Mesa & Morales, 1972)**

**Distribution.** Colombia*.

**Pintomyia (Pifanomyia) sauroida (Osorno-Mesa, Morales & Osorno, 1972)**

**Distribution.** Colombia*, Venezuela.

**Pintomyia (Pifanomyia) spinicrassa (Morales, Osorno-Mesa, Osorno & Hoyos, 1969)**

**Distribution.** Colombia*, Venezuela.

**Pintomyia (Pifanomyia) torvida (Young, Morales & Ferro, 1994)**

**Distribution.** Colombia*.

**Pintomyia (Pifanomyia) townsendi (Ortiz, 1959)**

**Distribution.** Colombia, Venezuela*.

**Pintomyia (Pifanomyia) youngi (Feliciangeli & Murillo, 1985)**

**Distribution.** Colombia, Costa Rica, Venezuela*.

**Series verrucarum Fairchild, 1955**

**Pintomyia (Pifanomyia) andina (Osorno, Osorno-Mesa & Morales, 1972)**

**Distribution.** Colombia*.

**Pintomyia (Pifanomyia) antioquiensis Wolff & Galati, 2002**

**Distribution.** Colombia*.

**Pintomyia (Pifanomyia) aulari (Feliciangeli, Ordoñez & Manzanilla, 1984)**

**Distribution.** Venezuela*.

**Pintomyia (Pifanomyia) cajamarcensis (Galati, Cáceres & Le Pont, 1995)**

**Distribution.** Peru*.

**Pintomyia (Pifanomyia) columbiana (Ristorcelli & Van Ty, 1941)**

**Distribution.** Colombia*.

**Pintomyia (Pifanomyia) deorsa (Pérez, Ogusuku, Monje & Young, 1991)**

**Distribution.** Peru*.

**Pintomyia (Pifanomyia) disjuncta (Morales, Osorno & Osorno-Mesa, 1974)**

**Distribution.** Colombia*.

**Pintomyia (Pifanomyia) itza Ibáñez-Bernal, May-UC & Rebollar-Tellez, 2010**

**Distribution.** Mexico*.

**Pintomyia (Pifanomyia) moralesi (Young, 1979)**

**Distribution.** Colombia*.

**Pintomyia (Pifanomyia) verrucarum (Townsend, 1913)**

**Distribution.** Peru*.

**Subgenus (*Pintomyia*) Costa Lima, 1932**

**Pintomyia (Pintomyia) bianchigalatiae (Andrade-Filho, Aguiar, Dias & Falcão, 1999)**

**Distribution.** Argentina, Brazil*.

**Pintomyia (Pintomyia) christenseni (Young & Duncan, 1994)**

**Distribution.** Brazil, Colombia, Panama*, Trinidad and Tobago, Venezuela.

**Pintomyia (Pintomyia) damascenoi (Mangabeira, 1941)**

**Distribution.** Brazil*, Colombia, French Guiana, Surinam.

**Pintomyia (Pintomyia) fischeri (Pinto, 1926)**

**Distribution.** Argentina, Bolivia, Brazil*, Paraguay, Peru, Venezuela.

**Pintomyia (Pintomyia) gibsoni (Pifano & Ortiz, 1972)**

**Distribution.** Venezuela*.

**Pintomyia (Pintomyia) kuscheli (Le Pont, Martinez, Torrez-Espejo & Dujardin, 1998)**

**Distribution.** Bolivia*, Brazil.

**Pintomyia (Pintomyia) mamedei (Oliveira, Afonso, Dias & Brazil, 1994)**

**Distribution.** Brazil*.

**Pintomyia (Pintomyia) pessoai (Coutinho & Barretto, 1940)**

**Distribution.** Argentina, Brazil*, Paraguay.

***Pintomyia incertae sedis***

† ***Pintomyia adiketis* Poinar, 2008**

**Distribution.** Dominican amber*.

† ***Pintomyia bolontikui* Ibáñez-Bernal, Kraemer, Stebner & Wagner, 2013**

**Distribution.** Mexican amber*.

† ***Pintomyia brazilorum* Andrade-Filho, Galati & Falcão, 2006**

**Distribution.** Dominican amber*

***Pintomyia diamantinensis* (Barata, Serra e Meira & Carvalho, 2012)**

**Distribution.** Brazil*.

† ***Pintomyia dissimilis* Andrade-Filho, Serra e Meira, Sanguinette & Brazil, 2009**

**Distribution.** Dominican amber*

†***Pintomyia dominicana* Andrade-Filho, Galati & Brazil**, **2009**

**Distribution.** Dominican amber*

† ***Pintomyia falcaorum* Brazil & Andrade-Filho, 2002**

**Distribution.** Dominican amber*

† ***Pintomyia filipalpis* (Peñalver & Grimaldi, 2005)**

**Distribution.** Dominican amber*

† ***Pintomyia killickorum* Andrade-Filho & Brazil, 2004**

**Distribution.** Dominican amber*

***Pintomyia maracayensis* (Nuñez-Tovar, 1924)**

**Distribution.** Venezuela*.

† ***Pintomyia miocena* (Peñalver & Grimaldi, 2005)**

**Distribution.** Dominican amber*

***Pintomyia naiffi* (Freitas & Oliveira, 2013)**

**Distribution.** Brazil*.

***Pintomyia nuneztovari* (Ortiz, 1954)**

**Distribution.** Venezuela*.

† ***Pintomyia paleopestis* (Peñalver & Grimaldi, 2005)**

**Distribution.** Dominican Republic*.

***Pintomyia rangeliana* (Ortiz, 1953)**

**Distribution.** Colombia, Panama,Trinidad and Tobago, Venezuela*.

† ***Pintomyia succini* (Peñalver & Grimaldi, 2005)**

**Distribution.** Dominican amber*

**Genus *Dampfomyia* Addis, 1945**

**Subgenus (*Coromyia*) Barretto, 1962**

**Dampfomyia (Coromyia) aquilonia (Fairchild & Harwood, 1961)**

**Distribution.** Canada, United States of America*.

**Dampfomyia (Coromyia) beltrani (Vargas & Díaz-Nájera, 1951)**

**Distribution.** Honduras, Mexico*.

**Dampfomyia (Coromyia) deleoni (Fairchild & Hertig, 1947)**

**Distribution.** Belize, Costa Rica, El Salvador, Guatemala*, Honduras, Mexico.

**Dampfomyia (Coromyia) disneyi (Williams, 1987)**

**Distribution.** Belize*, Guatemala, Mexico.

**Dampfomyia (Coromyia) isovespertilionis (Fairchild & Hertig, 1958)**

**Distribution.** Colombia, Costa Rica, Panama*.

**Dampfomyia (Coromyia) steatopyga (Fairchild & Hertig, 1958)**

**Distribution.** Mexico*.

**Dampfomyia (Coromyia) vesicifera (Fairchild & Hertig, 1947)**

**Distribution.** Costa Rica, Nicaragua, Panama*.

**Dampfomyia (Coromyia) vespertilionis (Fairchild & Hertig, 1947)**

**Distribution.** Colombia, Costa Rica, Ecuador, Nicaragua, Panama*.

**Dampfomyia (Coromyia) viriosa (Fairchild & Hertig, 1958)**

**Distribution.** Costa Rica, Panama*.

**Dampfomyia (Coromyia) zeledoni (Young & Murillo, 1984)**

**Distribution.** Costa Rica*, Honduras, Nicaragua.

**Subgenus (*Dampfomyia*) Addis, 1945**

**Dampfomyia (Dampfomyia) anthophora (Addis, 1945)**

**Distribution.** Mexico, Nicaragua, United States of America*.

**Dampfomyia (Dampfomyia) atulapai (León, 1971)**

**Distribution.** El Salvador, Guatemala*, Mexico.

**Dampfomyia (Dampfomyia) dodgei (Vargas & Diaz-Nájera, 1953)**

**Distribution.** El Salvador, Mexico*.

**Dampfomyia (Dampfomyia) insolita (Fairchild & Hertig, 1956)**

**Distribution.** Costa Rica, Panama*.

**Dampfomyia (Dampfomyia) leohidalgoi (Ibáñez-Bernal, Hernández-Xoliot & Mendoza, 2006)**

**Distribution.** Mexico*.

**Dampfomyia (Dampfomyia) permira (Fairchild & Hertig, 1956)**

**Distribution.** Belize, Guatemala, Mexico*.

**Dampfomyia (Dampfomyia) rosabali (Fairchild & Hertig, 1956)**

**Distribution.** Colombia, Costa Rica, Panama*.

**Group delpozoi Young & Fairchild, 1974**

***Dampfomyia delpozoi* (Vargas & Díaz-Nájera, 1953)**

**Distribution.** Belize, Guatemala, Mexico*.

***Dampfomyia inusitata* (Fairchild & Hertig, 1961)**

**Distribution.** Mexico*.

**Note**. The *Dampfomyia delpozoi* group shows characters of both the subgenus Coromyia and *Dampfomyia* *s.str.* and are therefore listed separately.

***Dampfomyia incertae sedis***

***Dampfomyia caminoi* (Young & Duncan, 1994)**

**Distribution.** Mexico*.

**Genus *Expapillata* Galati, 1995**

***Expapillata cerradincola* (Galati, Nunes, Oshiro & Dorval, 1995)**

**Distribution.** Brazil*.

***Expapillata firmatoi* (Barretto, Martins & Pellegrino, 1956)**

**Distribution.** Argentina, Brazil*.

**Genus *Pressatia* Mangabeira, 1942**

***Pressatia calcarata* (Martins & Silva, 1964)**

**Distribution.** Bolivia, Brazil*, Peru, Venezuela.

***Pressatia camposi* (Rodríguez, 1950)**

**Distribution.** Colombia, Costa Rica, Ecuador*, Nicaragua, Panama.

***Pressatia choti* (Floch & Abonnenc, 1941)**

**Distribution.** Bolivia, Brazil, Colombia, Ecuador, French Guiana*, Peru, Surinam.

***Pressatia duncanae* (Le Pont, Martinez, Torrez-Espejo & Durjardin, 1998)**

**Distribution.** Bolivia*, Colombia, Peru.

**Note.** The record of this species for Peru is from the illustrations published by Velasco 1973: 88 (*Lutzomyia* sp. D) and Young & Morales 1987: 662 (*Lutzomyia* sp. 1).

***Pressatia dysponeta* (Fairchild & Hertig, 1952)**

**Distribution.** Brazil, Colombia, Costa Rica, Ecuador, Panama*, Venezuela.

***Pressatia equatorialis* (Mangabeira, 1942)**

**Distribution.** Brazil*, French Guiana.

***Pressatia triacantha* (Mangabeira, 1942)**

**Distribution.** Brazil*, Colombia, Ecuador, French Guiana, Peru, Venezuela.

***Pressatia trispinosa* (Mangabeira, 1942)**

**Distribution.** Brazil*, French Guiana, Peru.

**Genus *Trichopygomyia* Barretto, 1962**

***Trichopygomyia conviti* (Ramírez-Pérez, Martins & Ramírez, 1976)**

**Distribution.** Brazil, Colombia, Venezuela*.

***Trichopygomyia dasypodogeton* (Castro, 1939)**

**Distribution.** Bolivia, Brazil*.

***Trichopygomyia depaquiti* (Gantier, Gaborit & Rabarison, 2006)**

**Distribution.** Brazil, French Guiana*.

***Trichopygomyia elegans* (Martins, Llanos & Silva, 1976)**

**Distribution.** Brazil, Peru*.

***Trichopygomyia ferroae* (Young & Morales, 1987)**

**Distribution.** Colombia*.

***Trichopygomyia gantieri* (Le Pont & Desjeux, 1987)**

**Distribution.** Bolivia*.

***Trichopygomyia longispina* (Mangabeira, 1942)**

**Distribution.** Brazil*, Colombia, French Guiana, Venezuela.

***Trichopygomyia martinezi* (Young & Morales, 1987)**

**Distribution.** Colombia*.

***Trichopygomyia pinna* (Feliciangeli, Ramírez-Pérez & Ramírez, 1989)**

**Distribution.** Brazil, Venezuela*.

***Trichopygomyia ratcliffei* (Arias, Ready & Freitas, 1983)**

**Distribution.** Brazil*.

***Trichopygomyia rondoniensis* (Martins, Falcão & Silva, 1965)**

**Distribution.** Bolivia, Brazil*.

***Trichopygomyia trichopyga* (Floch & Abonnenc, 1945)**

**Distribution.** Brazil, French Guiana*, Surinam.

***Trichopygomyia triramula* (Fairchild & Hertig, 1952)**

**Distribution.** Belize, Colombia, Costa Rica, Ecuador, Guatemala, Mexico, Panama*.

***Trichopygomyia turelli* (Fernández, Galati, Carbajal & Watts, 1998)**

**Distribution.** Peru*.

***Trichopygomyia wagleyi* (Causey & Damasceno, 1945)**

**Distribution.** Bolivia, Brazil*, Colombia, Venezuela.

***Trichopygomyia witoto* (Young & Morales, 1987)**

**Distribution.** Colombia*, Ecuador.

**Genus *Evandromyia* Mangabeira, 1941**

**Subgenus (*Aldamyia*) Galati, 1995**

**Evandromyia (Aldamyia) aldafalcaoae (Santos, Andrade-Filho & Honer, 2001)**

**Distribution.** Brazil*.

**Evandromyia (Aldamyia) andersoni (Le Pont & Desjeux, 1988)**

**Distribution.** Bolivia*, Brazil.

**Evandromyia (Aldamyia) apurinan Shimabukuro, Figueira & Silva, 2013**

**Distribution.** Brazil*.

**Evandromyia (Aldamyia) bacula (Martins, Falcão & Silva, 1965)**

**Distribution.** Bolivia, Brazil*.

**Evandromyia (Aldamyia) carmelinoi (Ryan, Fraiha, Lainson & Shaw, 1986)**

**Distribution.** Brazil*.

**Evandromyia (Aldamyia) dubitans (Sherlock, 1962)**

**Distribution.** Brazil, Colombia*, Costa Rica, Panama, Trinidad and Tobago, Venezuela.

**Evandromyia (Aldamyia) evandroi (Costa Lima & Antunes, 1936)**

**Distribution.** Argentina, Brazil*.

**Evandromyia (Aldamyia) hashiguchii León, Teran, Neira & Le Pont, 2009**

**Distribution.** Ecuador*.

**Evandromyia (Aldamyia) lenti (Mangabeira, 1938)**

**Distribution.** Bolivia, Brazil*, Surinam.

**Evandromyia (Aldamyia) orcyi Oliveira, Sanguinette, Almeida & Andrade-Filho, 2015**

**Distribution.** Brazil*.

**Evandromyia (Aldamyia) sericea (Floch & Abonnenc, 1944)**

**Distribution.** Brazil, Colombia, Ecuador, French Guiana*, Surinam, Venezuela.

**Evandromyia (Aldamyia) termitophila (Martins, Falcão & Silva, 1964)**

**Distribution.** Bolivia, Brazil*.

**Evandromyia (Aldamyia) walkeri (Newstead, 1914)**

**Distribution.** Bolivia*, Brazil*, Colombia, Ecuador, French Guiana, Panama, Peru, Trinidad and Tobago, Venezuela.

**Note**. The type locality of *Evandromyia walkeri* is along the Abuña river, which forms part of the border between Bolivia and Brazil, however it is not clear in which of the countries the exact type locality is located.

**Evandromyia (Aldamyia) williamsi (Damasceno, Causey & Arouck, 1945)**

**Distribution.** Brazil*, Peru, Venezuela.

**Subgenus (*Barrettomyia*) Martins and Silva, 1968**

**Series cortelezzii Galati, 1995**

**Evandromyia (Barrettomyia) cortelezzii (Brèthes, 1923)**

**Distribution.** Argentina*, Bolivia, Brazil, Paraguay, Peru, Uruguay.

**Evandromyia (Barrettomyia) corumbaensis (Galati, Nunes, Oshiro & Rego, 1989)**

**Distribution.** Bolivia, Brazil*.

**Evandromyia (Barrettomyia) sallesi (Galvão & Coutinho, 1939)**

**Distribution.** Argentina, Bolivia, Brazil*, Ecuador, Paraguay, Peru.

**Evandromyia (Barrettomyia) spelunca Carvalho, Brazil, Sanguinette & Andrade-Filho, 2011**

**Distribution.** Brazil*.

**Series monstruosa Lewis, Young & Minter, 1977**

**Evandromyia (Barrettomyia) monstruosa (Floch & Abonnenc, 1944)**

**Distribution.** Brazil, Colombia, French Guiana*, Surinam, Venezuela.

**Evandromyia (Barrettomyia) teratodes (Martins, Falcão & Silva, 1964)**

**Distribution.** Brazil*, Paraguay.

**Series tupynambai Martins & Silva, 1968**

**Evandromyia (Barrettomyia) bahiensis (Mangabeira & Sherlock, 1961)**

**Distribution.** Brazil*.

**Evandromyia (Barrettomyia) callipyga (Martins & Silva, 1965)**

**Distribution.** Brazil*.

**Evandromyia (Barrettomyia) costalimai (Mangabeira, 1942)**

**Distribution.** Brazil*.

**Evandromyia (Barrettomyia) petropolitana (Martins & Silva, 1968)**

**Distribution.** Brazil*.

**Evandromyia (Barrettomyia) tupynambai (Mangabeira, 1942)**

**Distribution.** Brazil*.

**Subgenus (*Evandromyia*) Mangabeira, 1941**

**Series infraspinosa Young & Arias, 1977**

**Evandromyia (Evandromyia) begonae (Ortiz & Torrez, 1975)**

**Distribution.** Brazil, Colombia, Venezuela*.

**Evandromyia (Evandromyia) bourrouli (Barretto & Coutinho, 1941)**

**Distribution.** Bolivia, Brazil*.

**Evandromyia (Evandromyia) brachyphalla (Mangabeira, 1941)**

**Distribution.** Brazil*, French Guiana.

**Evandromyia (Evandromyia) georgii (Freitas & Barrett, 2002)**

**Distribution.** Brazil*.

**Evandromyia (Evandromyia) infraspinosa (Mangabeira, 1941)**

**Distribution.** Bolivia, Brazil*, Colombia, French Guiana, Peru, Surinam, Venezuela.

**Evandromyia (Evandromyia) inpai (Young & Arias, 1977)**

**Distribution.** Brazil*, Venezuela.

**Evandromyia (Evandromyia) ledezmaae León, Teran, Neira & Le Pont, 2009**

**Distribution.** Ecuador*.

**Evandromyia (Evandromyia) pinottii (Damasceno & Arouck, 1956**)

**Distribution.** Brazil*, French Guiana, Venezuela.

**Evandromyia (Evandromyia) sipani (Fernández, Carbajal, Alexander & Need, 1994)**

**Distribution.** Brazil, Colombia, Peru*.

**Evandromyia (Evandromyia) tarapacaensis (Le Pont, Torrez-Espejo & Galati, 1997)**

**Distribution.** Bolivia*, Brazil.

**Series rupicola Young & Fairchild, 1974**

**Evandromyia (Evandromyia) correalimai (Martins, Coutinho & Luz, 1970)**

**Distribution.** Brazil*.

**Evandromyia (Evandromyia) gaucha Andrade-Filho, Souza & Falcão, 2007**

**Distribution.** Brazil*.

**Evandromyia (Evandromyia) grimaldii Andrade-Filho, Pinto, Santos & Carvalho, 2009**

**Distribution.** Brazil*.

**Evandromyia (Evandromyia) rupicola (Martins, Godoy & Silva, 1962)**

**Distribution.** Brazil*.

**Evandromyia (Evandromyia) tylophalla Andrade & Galati, 2012**

**Distribution.** Brazil*.

**Series saulensis Lewis, Young & Minter, 1977**

**Evandromyia (Evandromyia) saulensis (Floch & Abonnenc, 1944)**

**Distribution.** Bolivia, Brazil, Colombia, Costa Rica, Ecuador, French Guiana*, Panama, Peru, Venezuela.

**Evandromyia (Evandromyia) wilsoni (Damasceno & Causey, 1945)**

**Distribution.** Brazil*.

***Evandromyia incertae sedis***

***Evandromyia edwardsi* (Mangabeira, 1941)**

**Distribution.** Brazil*.

**Subtribe Psychodopygina Galati, 1995**

**Genus *Psathyromyia* Barretto, 1962**

**Subgenus (*Forattiniella*) Vargas, 1978**

**Psathyromyia (Forattiniella) abunaensis (Martins, Falcão & Silva, 1965)**

**Distribution.** Bolivia, Brazil*, Colombia, Ecuador, Peru.

**Psathyromyia (Forattiniella) antezanai (Le Pont, Dujardin, Mouchet & Desjeux, 1990)**

**Distribution.** Bolivia*.

**Psathyromyia (Forattiniella) aragaoi (Costa Lima, 1932)**

**Distribution.** Bolivia, Brazil*, Colombia, Costa Rica, Ecuador, French Guiana, Panama, Paraguay, Peru, Trinidad and Tobago, Venezuela.

**Psathyromyia (Forattiniella) barrettoi barrettoi (Mangabeira, 1942)**

**Distribution.** Bolivia, Brazil*, Colombia, Ecuador, French Guiana, Peru, Surinam, Trinidad and Tobago.

**Psathyromyia (Forattiniella) barrettoi majuscula (Young, 1979)**

**Distribution.** Colombia, Costa Rica, Ecuador, El Salvador, Honduras, Nicaragua, Panama*.

**Psathyromyia (Forattiniella) brasiliensis (Costa Lima, 1932)**

**Distribution.** Brazil*, French Guiana, Peru.

**Psathyromyia (Forattiniella) campograndensis (Oliveira, Andrade-Filho, Falcão & Brazil, 2001)**

**Distribution.** Brazil*, French Guiana.

**Psathyromyia (Forattiniella) carpenteri (Fairchild & Hertig, 1953)**

**Distribution.** Belize, Colombia, Costa Rica, Mexico, Panama*.

**Psathyromyia (Forattiniella) castilloi (León, Mollinedo & Le Pont, 2009)**

**Distribution.** Bolivia, Ecuador*, French Guiana.

**Psathyromyia (Forattiniella) coutinhoi (Mangabeira, 1942)**

**Distribution.** Bolivia, Brazil*, Peru.

**Psathyromyia (Forattiniella) elizabethdorvalae Brilhante, Sábio & Galati, 2016**

**Distribution.** Brazil*.

**Psathyromyia (Forattiniella) inflata (Floch & Abonnenc, 1944)**

**Distribution.** Bolivia, Brazil, French Guiana*.

**Psathyromyia (Forattiniella) lutziana (Costa Lima, 1932)**

**Distribution.** Bolivia, Brazil*, Colombia, French Guiana, Peru, Surinam, Venezuela.

**Psathyromyia (Forattiniella) naftalekatzi (Falcão, Andrade-Filho, Almeida & Brandão-Filho, 2000)**

**Distribution.** Brazil*.

**Psathyromyia (Forattiniella) pascalei (Coutinho & Barretto, 1940)**

**Distribution.** Brazil*.

**Psathyromyia (Forattiniella) pradobarrientosi (Le Pont, Matias, Martinez & Dujardin, 2004)**

**Distribution.** Bolivia*, Brazil.

**Note.** This species has been collected in Brazil in Amapá (PHFS) and in Distrito Federal (AJA).

**Psathyromyia (Forattiniella) runoides (Faichild & Hertig, 1953)**

**Distribution.** Brazil, Colombia, Costa Rica, Ecuador, Panama*, Peru.

†**Psathyromyia (Forattiniella) schleei (Peñalver & Grimaldi, 2005)**

**Distribution.** Dominican amber*

**Psathyromyia (Forattiniella) texana (Dampf, 1938)**

**Distribution.** Mexico*, United States of America.

**Subgenus (*Psathyromyia*) Barretto, 1962**

**Series lanei Theodor, 1965**

**Psathyromyia (Psathyromyia) digitata (Damasceno & Arouck, 1950)**

**Distribution.** Brazil*.

**Psathyromyia (Psathyromyia) lanei (Barretto & Coutinho, 1941)**

**Distribution.** Argentina, Brazil*, Paraguay.

**Psathyromyia (Psathyromyia) pelloni (Sherlock & Alencar, 1959)**

**Distribution.** Brazil*.

**Series shannoni Fairchild, 1955**

**Psathyromyia (Psathyromyia) abonnenci (Floch & Chassignet, 1947)**

**Distribution.** Bolivia, Brazil, Colombia, Ecuador, French Guiana*, Panama, Peru, Surinam, Venezuela.

**Psathyromyia (Psathyromyia) baratai Sábio, Andrade & Galati, 2015**

**Distribution.** Brazil*.

**Psathyromyia (Psathyromyia) barretti Alves & Freitas, 2016**

**Distribution.** Brazil*.

**Psathyromyia (Psathyromyia) bigeniculata (Floch & Abonnenc, 1941)**

**Distribution.** Brazil, French Guiana*.

**Psathyromyia (Psathyromyia) campbelli (Damasceno, Causey & Arouck, 1945)**

**Distribution.** Bolivia, Brazil*, Colombia, French Guiana, Peru, Venezuela.

**Psathyromyia (Psathyromyia) cratifer (Fairchild & Hertig, 1961)**

**Distribution.** Belize, Costa Rica, Honduras, Mexico*, Panama.

**Psathyromyia (Psathyromyia) dasymera (Fairchild & Hertig, 1961)**

**Distribution.** Belize, Brazil, Colombia, Costa Rica, Ecuador, Mexico, Nicaragua, Panama*, Venezuela.

**Psathyromyia (Psathyromyia) dendrophyla (Mangabeira, 1942)**

**Distribution.** Bolivia, Brazil*, Colombia, Ecuador, French Guiana, Peru, Surinam, Venezuela.

**Psathyromyia (Psathyromyia) guatemalensis (Porter & Young, 1986)**

**Distribution.** Guatemala*.

**Psathyromyia (Psathyromyia) lerayi (Le Pont, Martinez, Torrez-Espejo & Dujardin, 1998)**

**Distribution.** Bolivia*, Colombia.

**Psathyromyia (Psathyromyia) limai (Fonseca, 1935)**

**Distribution.** Brazil*.

**Psathyromyia (Psathyromyia) pifanoi (Ortiz, 1972)**

**Distribution.** Brazil, Colombia, Peru*.

**Psathyromyia (Psathyromyia) punctigeniculata (Floch & Abonnenc, 1944)**

**Distribution.** Argentina, Bolivia, Brazil, Colombia, Ecuador, French Guiana*, Panama, Peru, Surinam, Venezuela.

**Psathyromyia (Psathyromyia) ribeirensis Sábio, Andrade & Galati, 2014**

**Distribution.** Brazil*.

**Psathyromyia (Psathyromyia) scaffi (Damasceno & Arouck, 1956)**

**Distribution.** Bolivia, Brazil*, Colombia, French Guiana, Peru, Surinam.

**Psathyromyia (Psathyromyia) shannoni (Dyar, 1929)**

**Distribution.** Belize, Bolivia, Colombia, Costa Rica, Ecuador, French Guiana, Guatemala, Honduras, Mexico, Nicaragua, Panama*, Peru, Surinam, Trinidad and Tobago, United States of America, Venezuela.

**Psathyromyia (Psathyromyia) soccula (Fairchild & Hertig, 1961)**

**Distribution.** Costa Rica, Panama*.

**Psathyromyia (Psathyromyia) souzacastroi (Damasceno & Causey, 1944)**

**Distribution.** Brazil*.

**Psathyromyia (Psathyromyia) undulata (Fairchild & Hertig, 1950)**

**Distribution.** Belize, Bolivia, Colombia, Costa Rica, Ecuador, El Salvador, French Guiana*, Guatemala, Honduras, Mexico, Panama.

**Psathyromyia (Psathyromyia) volcanensis (Fairchild & Hertig, 1950)**

**Distribution.** Bolivia, Costa Rica, Panama*.

**Subgenus (*Xiphopsathyromyia*) Ibáñez-Bernal & Marina, 2015**

**Psathyromyia (Xiphopsathyromyia) aclydifera (Fairchild & Hertig, 1952)**

**Distribution.** Belize, Bolivia, Colombia, Costa Rica, Ecuador, Guatemala, Honduras, Mexico, Nicaragua, Panama*.

**Psathyromyia (Xiphopsathyromyia) dreisbachi (Causey & Damasceno, 1945)**

**Distribution.** Bolivia, Brazil*, Colombia, Ecuador, French Guiana, Peru, Surinam, Venezuela.

**Psathyromyia (Xiphopsathyromyia) hermanlenti (Martins, Silva & Falcão, 1970)**

**Distribution.** Brazil*.

**Psathyromyia (Xiphopsathyromyia) ruparupa (Martins, Llanos & Silva, 1976)**

**Distribution.** Bolivia, Peru*.

***Psathyromyia incertae sedis***

***Psathyromyia maya* Ibáñez-Bernal, May-UC & Rebollar-Tellez, 2010**

**Distribution.** Mexico*.

**Genus *Viannamyia* Mangabeira, 1941**

***Viannamyia caprina* (Osorno-Mesa, Morales & Osorno, 1972)**

**Distribution.** Colombia*, Costa Rica, Honduras, Panama, Peru, Nicaragua.

***Viannamyia fariasi* (Damasceno, Causey & Arouck, 1945)**

**Distribution.** Brazil*, French Guiana.

***Viannamyia furcata* (Mangabeira, 1941)**

**Distribution.** Bolivia, Brazil*, Colombia, Costa Rica, Ecuador, French Guiana, Peru, Venezuela.

***Viannamyia tuberculata* (Mangabeira, 1941)**

**Distribution.** Bolivia, Brazil*, Colombia, French Guiana, Panama, Peru, Surinam, Venezuela.

**Genus *Martinsmyia* Galati, 1995**

**Group alphabetica Fairchild, 1955**

***Martinsmyia alphabetica* (Fonseca, 1936)**

**Distribution.** Argentina, Brazil*, Paraguay.

***Martinsmyia brisolai* (Le Pont & Desjeux, 1987)**

**Distribution.** Bolivia*, Brazil.

***Martinsmyia minasensis* (Mangabeira, 1942)**

**Distribution.** Brazil*.

***Martinsmyia mollinedoi* (Le Pont & Desjeux, 1991)**

**Distribution.** Bolivia*.

***Martinsmyia oliveirai* (Martins, Silva & Falcão, 1970)**

**Distribution.** Brazil*.

***Martinsmyia pisuquia* (Ogusuku, Guevara, Revilla, Inga & Pérez, 2001)**

**Distribution.** Peru*.

***Martinsmyia quadrispinosa* (Floch & Chassignet, 1947)**

**Distribution.** French Guiana*.

***Martinsmyia reginae* Carvalho, Brazil, Sanguinette & Andrade-Filho, 2010**

**Distribution.** Brazil*.

***Martinsmyia waltoni* (Arias, Freitas & Barrett, 1984)**

**Distribution.** Brazil*.

**Group gasparviannai Young & Fairchild, 1974**

***Martinsmyia cipoensis* (Martins, Falcão & Silva, 1964)**

**Distribution.** Brazil*.

***Martinsmyia gasparviannai* (Martins, Godoy & Silva, 1962)**

**Distribution.** Brazil*.

**Genus *Bichromomyia* Artemiev, 1991**

***Bichromomyia flaviscutellata* (Mangabeira, 1942)**

**Distribution.** Bolivia, Brazil*, Colombia, Ecuador, French Guiana, Peru, Surinam, Trinidad and Tobago, Venezuela.

***Bichromomyia inornata* (Martins, Falcão & Silva, 1965)**

**Distribution.** Bolivia, Brazil*.

**Note.** Several authors (Young and Arias 1982, Young and Duncan 1994, Carvalho et al. 2015) have mentioned the possibility that *Bichromomyia inornata* is conspecific with *Bichromomyia flaviscutellata*. According to the description of *Bichromomyia inornata*, its scutellum is dark, which would distinguish this species from all other species in the genus *Bichromomyia*. However, at FIOCRUZ/COLFLEB, we have checked the holotype (slide number 39.581) plus three males from Maranhão (slide numbers NE 1139.63, No. 32203; NE 930.62, No. 28734; NE 933.62, No. 28761) that were identified as *Bichromomyia inornata* by the authors of the species, and all specimens have pale rather than dark scutellum, making this species indistinguishable from *Bichromomyia flaviscutellata*.

***Bichromomyia olmeca bicolor* (Fairchild & Theodor, 1971)**

**Distribution.** Brazil, Colombia, Costa Rica, Ecuador, Panama*, Peru, Venezuela.

***Bichromomyia olmeca nociva* (Young & Arias, 1982)**

**Distribution.** Brazil*, Peru.

***Bichromomyia olmeca olmeca* (Vargas & Díaz-Nájera, 1959)**

**Distribution.** Belize, Costa Rica, Guatemala, Honduras, Mexico*, Nicaragua.

***Bichromomyia reducta* (Feliciangeli, Ramírez-Pérez & Ramírez, 1988)**

**Distribution.** Brazil, Colombia, Peru, Venezuela*.

**Genus *Psychodopygus* Mangabeira, 1941**

**Series arthuri Barretto, 1962**

***Psychodopygus arthuri* (Fonseca, 1936)**

**Distribution.** Brazil*.

***Psychodopygus lloydi* (Antunes, 1937)**

**Distribution.** Brazil*.

***Psychodopygus matosi* (Barretto & Zago, 1956)**

**Distribution.** Brazil*.

**Series chagasi Barretto, 1962**

***Psychodopygus bernalei* (Osorno-Mesa, Morales & Osorno, 1967)**

**Distribution.** Bolivia, Brazil, Colombia*, Venezuela.

***Psychodopygus chagasi* (Costa Lima, 1941)**

**Distribution.** Brazil*, Colombia, Peru, Venezuela.

***Psychodopygus complexus* (Mangabeira, 1941)**

**Distribution.** Bolivia, Brazil*.

***Psychodopygus douradoi* (Fé, Freitas & Barrett, 1998)**

**Distribution.** Brazil*.

***Psychodopygus fairtigi* (Martins, 1970)**

**Distribution.** Colombia*.

***Psychodopygus killicki* (Feliciangeli, Ramírez-Pérez & Ramírez, 1988)**

**Distribution.** Venezuela*.

***Psychodopygus leonidasdeanei* Fraiha, Ryan, Ward, Lainson & Shaw, 1986**

**Distribution.** Brazil*.

***Psychodopygus squamiventris maripaensis* (Floch & Abonnenc, 1946)**

**Distribution.** Brazil, French Guiana*, Surinam.

***Psychodopygus squamiventris squamiventris* (Lutz & Neiva, 1912)**

**Distribution.** Brazil*, French Guiana, Peru, Venezuela.

***Psychodopygus wellcomei* Fraiha, Shaw & Lainson, 1971**

**Distribution.** Brazil*, Venezuela.

**Series davisi Barretto, 1962**

***Psychodopygus amazonensis* (Root, 1934)**

**Distribution.** Bolivia, Brazil, Colombia, Ecuador, French Guiana, Peru*, Surinam, Trinidad and Tobago, Venezuela.

***Psychodopygus claustrei* (Abonnenc, Léger & Fauran, 1979)**

**Distribution.** Bolivia, Brazil, Colombia, French Guiana*, Peru, Surinam, Venezuela.

***Psychodopygus davisi* (Root, 1934)**

**Distribution.** Bolivia, Brazil*, Colombia, Ecuador, French Guiana, Peru, Surinam, Venezuela.

***Psychodopygus parimaensis* (Ortiz & Álvarez, 1972)**

**Distribution.** Venezuela*.

**Series guyanensis Barretto, 1962**

***Psychodopygus corossoniensis* (Le Pont & Pajot, 1978)**

**Distribution.** Brazil, Costa Rica, French Guiana*, Mexico, Panama, Surinam.

***Psychodopygus dorlinsis* (Le Pont & Desjeux, 1982)**

**Distribution.** French Guiana*.

***Psychodopygus francoisleponti* Zapata, Depaquit & León, 2012**

**Distribution.** Ecuador*.

***Psychodopygus geniculatus* (Mangabeira, 1941)**

**Distribution.** Belize, Bolivia, Brazil*, Colombia, Costa Rica, Ecuador, French Guiana, Guatemala, Panama, Peru, Nicaragua, Venezuela.

***Psychodopygus guyanensis* (Floch & Abonnenc, 1941)**

**Distribution.** Belize, Colombia, Ecuador, French Guiana*, Peru, Surinam.

***Psychodopygus lainsoni* (Fraiha & Ward, 1974)**

**Distribution.** Bolivia, Brazil*, Peru.

***Psychodopygus luisleoni* León, Mollinedo & Le Pont, 2009**

**Distribution.** Ecuador*.

**Series panamensis Young & Fairchild, 1974**

***Psychodopygus ayrozai* (Barretto & Coutinho, 1940)**

**Distribution.** Bolivia, Brazil*, Colombia, Ecuador, French Guiana, Panama Peru, Trinidad and Tobago, Venezuela.

***Psychodopygus carrerai* (Barretto, 1946)**

**Distribution.** Bolivia, Brazil, Colombia*, Ecuador, Peru, Venezuela.

***Psychodopygus fairchildi* (Barretto, 1966)**

**Distribution.** Brazil*.

***Psychodopygus hirsutus* (Mangabeira, 1942)**

**Distribution.** Bolivia, Brazil*, Colombia, Ecuador, French Guiana, Peru, Surinam.

***Psychodopygus hirsutus* (Mangabeira, 1942)**

**Distribution.** Bolivia, Brazil*, Colombia, Ecuador, French Guiana, Peru, Surinam.

***Psychodopygus joliveti* Le Pont, León, Galati & Dujardin, 2009**

**Distribution.** French Guiana*.

***Psychodopygus llanosmartinsi* Fraiha & Ward, 1980**

**Distribution.** Bolivia, Brazil, Peru*.

***Psychodopygus nicaraguensis* (Fairchild & Hertig, 1961)**

**Distribution.** Brazil, Panama, Nicaragua*.

***Psychodopygus nocticolus* (Young, 1973)**

**Distribution.** Bolivia, Colombia*, Ecuador, French Guiana, Mexico, Panama, Peru.

***Psychodopygus panamensis* (Shannon, 1926)**

**Distribution.** Belize, Brazil, Colombia, Costa Rica, Ecuador, French Guiana, Guatemala, Honduras, Mexico, Nicaragua, Panama*, Peru, Surinam, Venezuela.

***Psychodopygus paraensis* (Costa Lima, 1941)**

**Distribution.** Bolivia, Brazil*, Colombia, Ecuador, French Guiana, Peru, Surinam, Venezuela.

***Psychodopygus recurvus* (Young, 1973)**

**Distribution.** Colombia*, Panama.

***Psychodopygus thula* (Young, 1979)**

**Distribution.** Colombia, Costa Rica, Ecuador, Honduras, Panama*.

***Psychodopygus yasuniensis* León, Neira & Le Pont, 2009**

**Distribution.** Ecuador*.

***Psychodopygus yucumensis* (Le Pont, Caillard, Tibayrenc & Desjeux, 1986)**

**Distribution.** Bolivia*, Brazil, Peru.

***Psychodopygus incertae sedis***

***Psychodopygus bispinosus* (Fairchild & Hertig, 1951)**

**Distribution.** Belize, Brazil, Colombia, Costa Rica, Ecuador, French Guiana, Guatemala, Honduras, Mexico, Nicaragua, Panama*, Surinam.

**Genus *Nyssomyia* Barretto, 1962**

***Nyssomyia anduzei* (Rozeboom, 1942)**

**Distribution.** Brazil, Costa Rica, French Guiana, Panama, Peru, Venezuela*.

***Nyssomyia antunesi* (Coutinho, 1939)**

**Distribution.** Bolivia, Brazil*, Colombia, French Guiana, Peru, Surinam, Trinidad and Tobago, Venezuela.

***Nyssomyia bibinae* (Léger & Abonnenc, 1988)**

**Distribution.** French Guiana*.

***Nyssomyia delsionatali* Galati & Galvis, 2012**

**Distribution.** Brazil*.

***Nyssomyia edentula* (León, 1971)**

**Distribution.** Costa Rica, Guatemala*, Honduras, Panama.

***Nyssomyia elongata* (Floch & Abonnenc, 1945)**

**Distribution.** French Guiana*.

***Nyssomyia fraihai* (Martins, Falcão & Silva, 1979)**

**Distribution.** Bolivia, Brazil*, Peru.

***Nyssomyia hernandezi* (Ortiz, 1965)**

**Distribution.** Colombia, Venezuela*.

***Nyssomyia intermedia* (Lutz & Neiva, 1912)**

**Distribution.** Brazil*.

***Nyssomyia neivai* (Pinto, 1926)**

**Distribution.** Argentina, Bolivia, Brazil*, Paraguay.

***Nyssomyia pajoti* (Abonnenc, Léger & Fauran, 1979)**

**Distribution.** Brazil, Colombia, French Guiana*, Peru, Surinam.

***Nyssomyia richardwardi* (Ready & Fraiha, 1981)**

**Distribution.** Bolivia, Brazil*, Colombia, Ecuador, Peru.

***Nyssomyia shawi* (Fraiha, Ward & Ready, 1981)**

**Distribution.** Bolivia, Brazil*, Colombia, Peru.

***Nyssomyia sylvicola* (Floch & Abonnenc, 1945)**

**Distribution.** Brazil, French Guiana*.

***Nyssomyia trapidoi* (Fairchild & Hertig, 1952)**

**Distribution.** Colombia, Costa Rica, Ecuador, Guatemala, Hounduras, Nicaragua, Panama*.

***Nyssomyia umbratilis* (Ward & Fraiha, 1977)**

**Distribution.** Bolivia, Brazil*, Colombia, French Guiana, Peru, Surinam, Venezuela.

***Nyssomyia urbinatti* Galati & Galvis, 2012**

**Distribution.** Brazil*.

***Nyssomyia whitmani* (Antunes & Coutinho, 1939)**

**Distribution.** Argentina, Bolivia, Brazil*, French Guiana, Paraguay, Peru, Surinam.

***Nyssomyia ylephiletor* (Fairchild & Hertig, 1952)**

**Distribution.** Belize, Colombia, Costa Rica, Ecuador, Guatemala, Honduras, Mexico, Nicaragua, Panama*.

***Nyssomyia yuilli* (Young & Porter, 1972)**

**Distribution.** Bolivia, Brazil, Colombia*, Ecuador, Peru, Venezuela.

**Genus *Trichophoromyia* Barretto, 1962**

***Trichophoromyia acostai* (Llanos, 1966)**

**Distribution.** Peru*.

***Trichophoromyia adelsonsouzai* Santos, Silva, Barata, Andrade & Galati, 2013**

**Distribution.** Brazil*.

***Trichophoromyia arevaloi* Galati & Cáceres, 1999**

**Distribution.** Peru*.

***Trichophoromyia auraensis* (Mangabeira, 1942)**

**Distribution.** Bolivia, Brazil*, Colombia, Peru, Surinam, Venezuela.

***Trichophoromyia beniensis* (Le Pont & Desjeux, 1987)**

**Distribution.** Bolivia*.

***Trichophoromyia bettinii* (Feliciangeli, Ramírez-Pérez & Ramírez, 1988)**

**Distribution.** Colombia, Venezuela*.

***Trichophoromyia brachipyga* (Mangabeira, 1942)**

**Distribution.** Brazil*, French Guiana.

***Trichophoromyia castanheirai* (Damasceno, Causey & Arouck, 1945)**

**Distribution.** Brazil*.

***Trichophoromyia cellulana* (Young, 1979)**

**Distribution.** Colombia*, Ecuador.

***Trichophoromyia clitella* (Young & Pérez, 1994)**

**Distribution.** Brazil, Peru*.

***Trichophoromyia dunhami* (Causey & Damasceno, 1945)**

**Distribution.** Brazil*.

***Trichophoromyia eurypyga* (Martins, Falcão & Silva, 1963)**

**Distribution.** Brazil*, Venezuela.

***Trichophoromyia flochi* (Abonnenc & Chassignet, 1948)**

**Distribution.** Brazil, French Guiana*.

***Trichophoromyia gibba* (Young & Arias, 1994)**

**Distribution.** Brazil*.

***Trichophoromyia howardi* (Young, 1979)**

**Distribution.** Brazil, Colombia*, Peru.

***Trichophoromyia incasica* (Llanos, 1966)**

**Distribution.** Peru*.

***Trichophoromyia ininii* (Floch & Abonnenc, 1943)**

**Distribution.** Brazil, French Guiana*, Surinam.

***Trichophoromyia lopesi* (Damasceno, Causey & Arouck, 1945)**

**Distribution.** Brazil*.

***Trichophoromyia loretonensis* (Llanos, 1964)**

**Distribution.** Brazil, Peru*.

***Trichophoromyia meirai* (Causey & Damasceno, 1945)**

**Distribution.** Brazil*.

***Trichophoromyia melloi* (Causey & Damasceno, 1945)**

**Distribution.** Brazil*, Surinam.

***Trichophoromyia napoensis* (Young & Rogers, 1984)**

**Distribution.** Ecuador*.

***Trichophoromyia nautaensis* (Fernández, Lopez, Cardenas & Requena, 2015)**

**Distribution.** Peru*.

***Trichophoromyia nemorosa* (Young & Pérez, 1994)**

**Distribution.** Peru*.

***Trichophoromyia octavioi* (Vargas, 1949)**

**Distribution.** Bolivia, Brazil*, Peru.

***Trichophoromyia omagua* (Martins, Llanos & Silva, 1976)**

**Distribution.** Peru*.

***Trichophoromyia pabloi* (Barreto, Burbano & Young, 2002)**

**Distribution.** Colombia*, Ecuador.

***Trichophoromyia pastazaensis* (Fernández, Carbajal, Alexander & Need, 1993)**

**Distribution.** Peru*.

***Trichophoromyia readyi* (Ryan, 1986)**

**Distribution.** Brazil*.

***Trichophoromyia reburra* (Fairchild & Hertig, 1961)**

**Distribution.** Colombia, Costa Rica, Ecuador, Panama*.

***Trichophoromyia reinerti* (Young & Duncan, 1994)**

**Distribution.** Brazil*.

***Trichophoromyia rostrans* (Summers, 1912)**

**Distribution.** Brazil*.

***Trichophoromyia ruifreitasi* Oliveira, Teles, Medeiros, Camargo & Pessoa, 2015**

**Distribution.** Brazil*.

***Trichophoromyia ruii* (Arias & Young, 1982)**

**Distribution.** Brazil*, Colombia.

***Trichophoromyia saltuosa* (Young, 1979)**

**Distribution.** Colombia*.

***Trichophoromyia sinuosa* (Young & Duncan, 1994)**

**Distribution.** Peru*.

***Trichophoromyia ubiquitalis* (Mangabeira, 1942)**

**Distribution.** Bolivia, Brazil*, Colombia, Ecuador, French Guiana, Peru, Surinam, Venezuela.

***Trichophoromyia uniniensis* Ladeia-Andrade, Fé, Sanguinette & Andrade-Filho, 2014**

**Distribution.** Brazil*.

***Trichophoromyia velascoi* (Le Pont & Desjeux, 1992)**

**Distribution.** Bolivia*.

***Trichophoromyia viannamartinsi* (Sherlock & Guitton, 1970)**

**Distribution.** Brazil*.

***Trichophoromyia wilkersoni* (Young & Rogers, 1984)**

**Distribution.** Ecuador*.

### Unplaced genera of Phlebotomini

**Genus *Edentomyia* Galati, Andrade-Filho, Silva & Falcão, 2003**

***Edentomyia piauiensis* Galati, Andrade-Filho, Silva & Falcão, 2003**

**Distribution.** Brazil*.

***Nomina dubia* in New World Phlebotominae**

***Nyssomyia singularis* (Costa Lima, 1932)**

**Distribution.** Brazil*.

**Note.** This species is only known from the type specimen mounted in Canada Balsam medium. The specimen “cotype” is deposited in the Coleção Entomológica do Instituto Oswaldo Cruz (FIOCRUZ/CEIOC) (number of the slides: 1436–1439). The specimen was collected in 08-VIII-1902 by Adolpho Lutz in Juqueri (currently Mairiporã municipality) state of São Paulo, Brazil. One of us (AJA) studied the type and observed that the thorax is damaged, but it is possible to observe the colour of the paratergite and scutum, which is similar to species in the genus *Nyssomyia*. The spermathecae was dissected, but was not observed in any of the slides, so it is likely the spermathecae has oxized over time. The original description is insufficient for a positive identification, however the spermathecae as illustrated show the same number of rings as *Nyssomyia neivai* In the absence of evidence positively linking the two species, however, we prefer to consider *Nyssomyia singularis* as a *nomen dubium*.

### Available names but found to denote more than one taxon (availability of the name is not affected according to provisions of the ICZN, Articles 17.2 and 23.8)

***Phlebotomus breviductus* Barretto, 1950**

**Note.** Only known from the holotype and five females collected by Rangel et al. (1985). Andrade et al. (2013) examined the holotype and concluded that the head and wing of this specimen belong to a *Trichopygomyia* sp. specimen, whereas the thorax and abdomen belong to an anomalous specimen of *Nyssomyia umbratilis*.

***Phlebotomus oliverioi* Barretto & Coutinho, 1941**

**Note.** Andrade et al. (2014) examined the holotype and concluded that the head of this specimen belongs to a specimen of *Psychodopygus* while the wings, thorax and abdomen belong to another specimen, of the genus *Psathyromyia*.

### Unavailable names not meeting the requirements of the ICZN

**Micropygomyia (Sauromyia) sp. 2 of Araracuara (Morales & Minter, 1981)**

**Distribution.** Colombia*.

**Note.** Unavailable according to article 11.4 of the ICZN. This species has been described from both males and females, but the authors decided not to name it.

**Lutzomyia (Helcocyrtomyia) sp. of Pichinde Young, 1979**

**Distribution.** Colombia*.

**Note**. Unavailable according to article 11.4 of the ICZN. This species has been described from both males and females. Young (1979) and Young and Duncan (1994) stated that this species was closely related to Lutzomyia (Helcocyrtomyia) hartmanni and Lutzomyia (Helcocyrtomyia) *scorzai*, but noted that further studies were necessary before formally naming this species.

***Pintomyia* sp. of Anchicaya (Young, 1979)**

**Distribution.** Colombia*.

**Note**. Unavailable according to article 11.4 of the ICZN. This species has been described form a single male, but Young and Duncan (1994) stated that they were waiting for the collection of the female before formally naming this species.

***Dampfomyia* sp. of Suchitepequez (Young & Duncan, 1994)**

**Distribution.** Guatemala*.

**Note.** This species appears as an illustration in Young and Duncan (1994: 247), who stated it was the holotype of *Lutzomyia piedraferroi*. However, Galati (2003) interpreted their illustration as a different species, which differs markedly in the number and shape of the spines in the gonostyle from the original description by León (1971); she referred to the species as *Dampfomyia* sp. of Suchitepequez, and it awaits formal description.

***Pressatia* #1 Mangabeira, 1942**

**Distribution.** Bolivia*, Colombia.

**Note.** Unavailable according to article 11.4 of the ICZN. This species is listed by Young and Duncan (1994), and reported as being described form a single male by Velasco (unpublished). However, Young and Duncan (1994) stated that the latter was waiting for the collection of the female before formally naming this species.

**Evandromyia (Aldamyia) sp. of Baduel (Floch & Abonnenc, 1945)**

**Distribution.** Brazil, Colombia, French Guiana*, Surinam.

**Note.** Unavailable according to article 11.4 of the ICZN. This species has been described from both males and females. Although this species has been recorded in different publications, no attempt has been made so far to formally describe it.

***Psychodopygus* sp. of Trés Esquinas (Young, 1979)**

**Distribution.** Colombia*.

**Note.** Unavailable according to article 11.4 of the ICZN. This species has been described only from females. Because females of the series guyanensis are indistinguishable in morphology, it is not possible to know if this species has been previously described from a male.

***Trichophoromyia* sp. 1 of Araracuara (Morales & Minter, 1981)**

**Distribution.** Colombia*.

**Note.** Unavailable according to article 11.4 of the ICZN. This species has been described from both males and females. However, the authors were not sure if it was a variant of *Trichophoromyia howardi* Young, 1979, and hence decided not to name it.
